# EHMTI-0026. Neuroprolotherapy and acupuncture for clinical trial of acute and chronic migraine treatment

**DOI:** 10.1186/1129-2377-15-S1-G34

**Published:** 2014-09-18

**Authors:** R Schulman

**Affiliations:** 1 Physical Medicine and Rehabilitation, Virginia Commonwealth University, Richmond, USA

## Summary

Neuroprolotherapy and acupuncture for clinical trial of acute and chronic migraine treatment.

## Objectives

Formulation of protocol for clinical trial of neuroprolotherapy and acupuncture treatment of migraine, partially based upon treatment sites used in OnabotulinumtoxinA treatment of migraine.

## Background

Studies thus far have demonstrated that acupuncture is both effective, and cost effective in the treatment and prevention of migraine. Neuroprolotherapy and acupuncture are less costly, and are associated with fewer risks, than OnabotulinumtoxinA. No studies have been published that examine the effects of neuroprolotherapy on migraine.

This protocol will serve as a template for use in a multi-center clinical trial to be conducted at a later date.

## Methods

Key muscles in the treatment of migraine, using either OnabotulinumtoxinA or acupuncture, are the frontalis, the corrugator supercilii, procerus, temporalis, occipitalis, trapezius, and splenius capitis. These muscle locations correspond to classical acupuncture point locations as follows: Frontalis:GB14, corrugator supercilii :BL2, procerus :GB24.5, or Yin Tang, temporalis :GB 4,5,6,7, occipitalis GB20, splenius capitus:BL10, trapezius GB21.

Neuroprolotherapy utilizes similar locations, with emphasis on “Chronic Constriction Injuries of the Peptidergic Sensory System”. These locations are chosen for their location directly over sensory nerve exits, and often correspond to acupuncture points as well. These locations include the nerve exits and course of the infraorbital (ST1), subraorbital(BL2), subratrochlear(BL1), infratrochlear(ST2), zygomatico-facial (SI18), zygomatic-temoral (GB1), auriculo-temporal (SI17), mental(Jiachengjiang [M-HN-18]), buccal, lessor occipital (GB12,), greater auricular(SI16), greater occipital(GB20,19), auriculotemporal nerve (GB4, 5, 6, 7), posterior cutaneous branches of dorsal rami of C4, 5, 6 (BL10).

Clinical experience indicates that Neuroprolotherapy is able to abort chronic cycling migraine, theoretically by repair of the peptidergic sensory TRPV1 receptors via the antagonist effect of 5% dextrose. Acupuncture activates the default mode network, and regulates heart rate variability and autonomic tone.

A protocol will be formulated that utilizes these points, as well as the addition of secondary acupuncture points chosen to alleviate myofascial strain patterns disrupting structural tensegrity. Acupuncture points helpful in the alleviation of nausea will also be chosen.

## Results

To be determined.

## Conclusion

The protocol will create a reproducible template of neuroprolotherapy and acupuncture points that can be used to conduct a multicenter clinical trial examining the efficacy of the use of these methods in the treatment of migraine prophylaxis and acute treatment.

No conflict of interest.

